# Non-alcoholic fatty liver disease and incidence of microvascular complications of diabetes in patients with type 2 diabetes: a prospective cohort study

**DOI:** 10.3389/fendo.2023.1147458

**Published:** 2023-06-05

**Authors:** Niloofar Deravi, Fatemeh Dehghani Firouzabadi, Fatemeh Moosaie, Hassan Asadigandomani, Melika Arab Bafrani, Niyoosha Yoosefi, Amirhossein Poopak, Mohammad Dehghani Firouzabadi, Mohadeseh Poudineh, Soghra Rabizadeh, Ibrahim Kamel, Manouchehr Nakhjavani, Alireza Esteghamati

**Affiliations:** ^1^ Endocrinology and Metabolism Research Center (EMRC), Vali-Asr Hospital, School of Medicine, Tehran University of Medical Sciences, Tehran, Iran; ^2^ Student Research committee, School of Medicine, Shahid Beheshti University of Medical Sciences, Tehran, Iran; ^3^ Radiology and Imaging Sciences, Clinical Center, National Institutes of Health, Bethesda, MD, United States; ^4^ School of Medicine, Zanjan University of Medical Sciences, Zanjan, Iran

**Keywords:** type 2 diabetes, non-alcoholic fatty liver disease, diabetic neuropathy, diabetic retinopathy, diabetic nephropathy

## Abstract

**Objective:**

To investigate the association between non-alcoholic fatty liver disease (NAFLD) and liver enzymes with the incidence of microvascular complications (neuropathy, retinopathy, and nephropathy) in a cohort of Iranian patients with type 2 diabetes.

**Methods:**

For a total population of 3123 patients with type 2 diabetes, a prospective study was designed for 1215 patients with NAFLD and 1908 gender and age-matched control patients without NAFLD. The two groups were followed for a median duration of 5 years for the incidence of microvascular complications. The association between having NAFLD, the level of liver enzymes, aspartate aminotransferase to platelet ratio index (APRI), Fibrosis-4 (FIB-4) value, and the incidence risk of diabetic retinopathy, neuropathy, and nephropathy were assessed through logistic regression analysis.

**Results:**

NAFLD was found to be associated with incidence of diabetic neuropathy and nephropathy (Odds ratio: 1.338 (95% confidence interval: 1.091-1.640) and 1.333 (1.007-1.764), respectively). Alkaline-phosphatase enzyme was found to be associated with higher risks of diabetic neuropathy and nephropathy ((Risk estimate: 1.002 (95% CI: 1.001-1.003) and 1.002 (1.001-1.004), respectively)). Moreover, gamma-glutamyl transferase was associated with a higher risk of diabetic nephropathy (1.006 (1.002-1.009). Aspartate aminotransferase and alanine aminotransferase were inversely associated with the risk of diabetic retinopathy (0.989 (0.979-0.998) and 0.990 (0.983-0.996), respectively). Furthermore, ARPI_T (1), ARPI_T (2), and ARPI_T (3) were shown to be associated with NAFLD (1.440 (1.061-1.954), 1.589 (1.163-2.171), and 2.673 (1.925, 3.710), respectively). However, FIB-4 score was not significantly associated with risk of microvascular complications.

**Conclusion:**

Despite the benign nature of NAFLD, patients with type 2 diabetes should be always assessed for NAFLD to ensure early diagnosis and entry into proper medical care. Regular screenings of microvascular complications of diabetes is also suggested for these patients.

## Introduction

Non-alcoholic fatty liver disease (NAFLD) occurs commonly in patients with type 2 diabetes mellitus, with a prevalence of 55%–68% (1) due to the frequent occurrence of insulin resistance and obesity in patients with type 2 diabetes (2).

There is now growing evidence that independent of other known risk factors and especially in patients with type 2 diabetes, NAFLD, could be associated with an increased risk of macrovascular complications. Several observational studies and some meta-analyses have documented that NAFLD, especially its advanced forms, is strongly associated with fatal and non-fatal cardiovascular events, as well as with specific cardiac complications, including sub-clinical myocardial alteration and dysfunction, heart valve diseases and cardiac arrhythmias. Importantly, across various studies, these associations remained significant after adjustment for established cardiovascular risk factors and other confounders (3–7).

Furthermore, It has been shown that NAFLD increases the risks for the development of type 2 diabetes and/or its progression (8, 9). Therefore, NAFLD could increase the risk for type 2 diabetes organ-specific complications as well and consequently the incidence of type 2 diabetes complications such as nephropathy, retinopathy, and neuropathy with NAFLD is an emerging concept (10).

Several population-based studies have revealed different types of associations between the incidence of microvascular complications of type 2 diabetes and NAFLD. A report from India revealed increased prevalence of microvascular complications including nephropathy and neuropathy in patients with type 2 diabetes and fatty liver disease (10). However, a cross sectional study of the Korean population reported that prevalence of diabetic nephropathy and retinopathy were lower in patients with type 2 diabetes who had NAFLD (11). Conversely, Targher et al. (12, 13) reported that NAFLD is independently associated with an increased prevalence of both diabetic nephropathy and retinopathy in patients with type 2 diabetes. Altogether, convincing epidemiological evidence have supported a strong association between the presence and severity of NAFLD, and the risk of chronic microvascular diabetes complications (14).

Since results of the previous studies are controversial, once again the present study aimed to investigate the association of NAFLD, liver enzymes, and Fibrosis-4 (FIB-4) index [a non-invasive fibrosis scoring systems to checkup liver fibrosis (15, 16)] with the incidence of microvascular complications (neuropathy, retinopathy, and nephropathy) in a cohort of Iranian patients with type 2 diabetes.

## Materials and methods

### Study population

In this prospective cohort study, 3123 patients with a history of type 2 diabetes enrolled and were followed for median of 5 years. The participants had all previously attended the endocrinology clinic of Vali-Asr Hospital, a medical center affiliated with Tehran University of Medical Sciences. The study group was chosen based on comprehensive exclusion criteria which consisted of having a history of glaucoma, vitreous surgery, cataract on eye examination, kidney disease (Creatinine (Cr)> 2 mg/dl) or low estimated glomerular filtration rate (eGFR) <30 cc/min), hypothyroidism, familial hypercholesterolemia, liver dysfunction, epilepsy, and hemoglobinopathy. Women taking oral contraceptives or hormone replacement therapy and pregnant women were also excluded. Additionally, patients with type 1 diabetes, gestational diabetes, diabetes due to pancreatic cancer, pancreatitis and other metabolic conditions were excluded. Patients with a history of alcohol use, viral hepatitis, hepatotoxicity-inducing drugs usage, autoimmune hepatitis, and/or rapid weight loss were excluded from the study. Baseline biochemical tests of the patients such as cholesterol levels and other lipid and glycemic indices were measured (**Table 1**).

**Table 1:** Baseline characteristics of the study population based on the presence of fatty liver.

	With fatty liver				Without fatty liver				P-value
	total	female	male	p-value	total	female	male	p-value	
**Age (year)**	51.34 ± 12.86	52.02 ± 12.98	50.56 ± 12.70	**0.005**	57.54 ± 28.91	56.40 ± 34.87	59.59 ± 12.04	**0.008**	**<0.001**
**Diabetes duration (year)**	9.44 ± 7.58	9.76 ± 7.69	9.12 ± 7.47	0.068	9.50 ± 8.58	8.86 ± 8.29	10.58 ± 8.96	**<0.001**	0. 797
**SBP (mmHg)**	126.23 ± 27.17	125.12 ± 16.97	127.50 ± 35.40	**0.030**	128.98 ± 31.60	127.72 ± 17.39	131.26 ± 47.41	**0.007**	**0.001**
**DBP (mmHg)**	78.76 ± 8.75	78.41 ± 8.85	79.15 ± 8.63	**0.037**	75.61 ± 10.57	74.85 ± 10.73	76.99 ± 10.13	**<0.001**	**<0.001**
**FBS (mg/dL)**	139.40 ± 55.17	134.43 ± 52.56	145.14 ± 55.54	**<0.001**	144.45 ± 56.55	139.95 ± 56.94	152.60 ± 54.96	**<0.001**	**0.001**
**2hPP (mg/dL)**	192.03 ± 86.73	181.11 ± 85.14	203.82 ± 86.93	**<0.001**	190.31 ± 88.78	178.55 ± 85.18	211.40 ± 91.22	**<0.001**	0.506
**Hb AIC (%)**	7.05 ± 1.69	6.89 ± 1.66	7.23 ± 1.71	**<0.001**	7.51 ± 14.63	7.51 ± 18.20	7.51 ± 1.67	0.995	0.134
**Chl (mg/dL)**	186.94 ± 43.97	191.95 ± 43.10	181.14 ± 44.28	**<0.001**	172.41 ± 44.75	177.16 ± 44.64	163.79 ± 43.68	**<0.001**	**<0.001**
**HDL (mg/dL)**	44.85 ± 11.63	47.68 ± 11.55	41.58 ± 10.87	**<0.001**	46.11 ± 12.13	48.10 ± 12.57	42.51 ± 10.38	**<0.001**	**<0.001**
**LDL (mg/dL)**	107.55 ± 35.16	110.42 ± 35.32	104.23 ± 34.71	**<0.001**	97.23 ± 33.62	99.66 ± 34.13	92.85 ± 32.25	**<0.001**	**<0.001**
**TG (mg/dL)**	183.54 ± 119.48	175.03 ± 95.02	193.36 ± 142.02	**<0.001**	155.65 ± 90.17	156.32 ± 86.30	154.44 ± 96.84	0.619	**<0.001**
**Cr (mg/dL)**	0.98 ± 0.21	0.90 ± 0.20	1.07 ± 0.20	**<0.001**	0.97 ± 0.24	0.91 ± 0.21	1.09 ± 0.26	**<0.001**	0.147
**eGFR (ml/min)**	49.00 ± 23.67	49.20 ± 31.54	48.83 ± 18.04	0.981	50.45 ± 20.59	48.82 ± 23.46	51.22 ± 17.84	0.884	0.860
**UA (mg/dL)**	5.36 ± 1.95	5.09 ± 2.14	5.66 ± 1.70	**<0.001**	4.78 ± 2.07	4.58 ± 1.71	5.15 ± 2.57	**<0.001**	**<0.001**
**BMI (Kg/m^2^)**	32.37 ± 6.48	33.70 ± 7.10	30.74 ± 5.20	**<0.001**	28.67 ± 5.17	29.41 ± 5.63	27.54 ± 4.14	**<0.001**	**<0.001**
**AST**	28.79 ± 17.11	27.17 ± 14.20	29.45 ± 17.41	**0.001**	19.33 ± 10.70	18.80 ± 8.61	20.09 ± 12.01	**0.002**	**<0.001**
**ALT**	40.38 ± 24.62	35.19 ± 20.63	43.39 ± 24.14	**<0.001**	21.67 ± 11.65	20.82 ± 11.25	23.18 ± 9.68	**<0.001**	**<0.001**
**ALKP**	166.84 ± 82.75	174.56 ± 89.15	158.46 ± 74.34	**<0.001**	141. 41 ± 69.54	143.05 ± 68.80	138.50 ± 70.79	0.183	**<0.001**
**GGT**	38.54 ± 50.51	35.94 ± 53.82	40.99 ± 47.14	0.185	27.82 ± 25.08	24.29 ± 20.19	32.19 ± 29.52	**0.002**	**<0.001**
**Retinopathy**	157 (8.96)	82 (4.68)	75 (4.28)	0.616	235 (10.37)	126 (5.56)	109 (4.81)	**0.002**	0.134
**Neuropathy**	228 (17.62)	116 (8.96)	112 (8.66)	0.942	296 (14.47)	157 (7.67)	139 (6.80)	**0.001**	**0.015**
**Nephropathy**	108 (8.67)	51 (4.09)	57 (4.58)	0.420	126 (6.50)	52 (2.68)	74 (3.82)	**<0.001**	**0.022**

SBP, systolic blood pressure; DBP, diastolic blood pressure; FBS, fasting blood sugar; 2hPP, Two-Hour Postprandial Glucose; Hb AIC, hemoglobin A1C; Chl, cholesterol; HDL, High density lipoproteins; LDL, low density lipoproteins; TG, triglycerides, Cr, creatinine; eGFR, Estimated Glomerular Filtration Rate; UA, uric acid; BMI, body mass index; AST, aspartate aminotransferase; ALT, Alanine transaminase; ALKP, Alkaline phosphatase, GGT, gamma-glutamyl transferase.Bold values report statistically significant difference.

NAFLD was defined as the presence of definite hepatic steatosis on ultrasound scan in the absence of a secondary cause for hepatic steatosis. The participants were divided into two study groups based on the presence of NAFLD at the start of the study: 1215 patients with NAFLD and 1908 without NAFLD. The presence of NAFLD was diagnosed based on the observation of definite hepatic steatosis on abdominal ultrasound performed by an expert radiologist (i.e., grades 2 or 3 hepatic steatosis, defined based on marked and diffuse hepatic hyperechogenicity relative to the renal parenchyma, ultra-sound beam attenuations and/or poor or no visualization of the diaphragm and intrahepatic vessels/structures, with or without focal fatty sparing consistent with the evidence of severe hepatic steatosis). Three hundred and fifty (350) patients who developed NAFLD over the duration of the study were excluded. During the study period, the status of possible chronic causes of non-NAFLD were constantly recorded. Patients with positive serology tests for hepatitis B, and C viruses surface antigens, and other causes of chronic liver diseases such as autoimmune hepatitis, hemochromatosis, Wilson’s disease, primary biliary cirrhosis, and sclerosing cholangitis were excluded from the current study, based on physical examinations and blood tests (i.e. antinuclear and anti-smooth muscle antibody, iron studies, ceruloplasmin, and urinary copper). After obtaining written informed consent from the participants, the incidence of microvascular complications was investigated in both groups. The study was reviewed and approved by the ethics committee of Tehran University of Medical Sciences.

### Physical examinations

For each participant, baseline demographic data such as weight, height, blood pressure, diabetes duration, the usage of anti-hypertensive and lipid lowering drugs were recorded by trained medical staff. Patients’ weight was measured using a portable digital scale with a precision of 0.1 kg, after they were asked to wear light clothing. An inflexible measurement tape with a precision of 0.1 cm was used to measure height with the subjects being asked to stand erect and remove their socks and shoes. Using the height and weight data, each individual’s body mass index (BMI) was then measured (Kg/m^2^). The subjects’ blood pressure was measured three times, after a ten-minute seated rest and within five-minute intervals. To measure blood pressure, a calibrated Omron M7 digital sphyg142 manometer (Hoofddorp, The Netherlands) with appropriately sized cuffs which covered at least 80% of the subjects’ right arm was used. First reading was discarded due to possible imprecision and the second and third readings were averaged to calculate the mean value of systolic (SBP) and diastolic blood pressure (DBP). The participants were asked to stand still in a relaxed position, placing both feet together on a flat surface for waist circumference (WC) measurements; one layer of clothing was accepted. A non-stretchable measuring tape was used to measure WC as the smallest horizontal girth between the costal margins and the iliac crest at minimal respiration. During the interview process, demographic information, smoking habits, and medication usage status were obtained.

### Laboratory analysis

Ten ml of blood sample was drawn from each individual who was asked to fast for 12 to 14 hours over night. The samples were kept at a temperature of 4 to 8 °C in cold biochemistry tubes and were later sent to the appropriate calibrating laboratories where they were instantly centrifuged (1500 rmp, for 10 min, at standard room temperature of 21 °C). The extracted serum was stored in the temperature of -70) (17). Laboratory evaluations were done on the extracted serum stored at a temperature of -70°C. In randomly selected urine samples, the measure of urinary albumin excretion was performed using urinary albumin-to-creatinine ratio (UACR). Urinary albumin concentrations were evaluated by an immunoturbidimetric commercial kit (Randox, Antrim, UK). The Chronic Kidney Disease Epidemiology Collaboration Equation was used to calculate the estimated glomerular filtration rate (eGFR).

Employing high-performance liquid chromatography (A1C, DS5 Pink kit; Drew, Marseille, France), Glycated hemoglobin (HbA1c) was measured. Fasting plasma glucose (FPG) and two-hour postprandial (2HPP) glucose were measured using colorimetric methods by the glucose oxidase test. Serum lipid concentrations [triglycerides(TG), high-density lipoprotein cholesterol (HDL-c), low-density lipoprotein cholesterol (LDL-c) (18), and total cholesterols (Chl) (19)] were measured using enzymatic methods. The kits used in this study were approved by the central reference laboratory in Tehran, Iran (17). Enzymatic photometry was used to analyze serum liver enzymes: alanine aminotransferase (ALT), aspartate aminotransferase (AST), alkaline phosphatase (ALKP), and gamma glutamyl transferase (GGT). Based on respective standardized criteria, an ALT level >30 IU/L in women and >40 IU/L in men, AST level >30 IU/L in women and >36 IU/L in men, ALKP levels of greater than 306 U/L in both genders and GGT levels greater than 60 U/L for men and greater than 40 IU/L in women were considered elevated. IFCC (International Federation of Clinical Chemistry and LaboratoryMedicine) method was employed to measure levels of ALT, AST and GGT (ALT intra-assay CV = 3.7%, AST intra-assay = 2.5% and GGT intra-assay CV = 2.2%) (20). The level of ALKP was measured according to Deutsche Gesellschaft für Klinische Chemie (DGKC) method (21). The liver enzyme measurements were performed using commercial Parsazmun kits (Tehran, Iran) and Hitachi 704 automatic analyzer (Tokyo, Japan).

A non-invasive diagnostic test for NAFLS is aspartate aminotransferase to platelet ratio index (APRI) which is calculated as (AST level/AST upper level of normal/platelet count) × 100 (22).

The FIB-4 index, a marker of hepatic fibrosis, was calculated by the following formula:

{age [years] × AST [U/L]/[platelet count (10^9^/L) × ALT (U/L)^1/2^]} (23)

### Assessment of complications

To identify the microvascular complications associated with diabetes, the International Classification of Diseases, Tenth Revision (ICD-10) was used. Diabetic neuropathy was identified using specific codes E10.4, E11.4, E12.4, E13.4, and E14.4. Diabetes-related chronic microvascular complications were identified according to the International Classification of Diseases, Tenth Revision (ICD-10). The specific codes used were: E10.3, E11.3, E12.3, E13.3 and E14.3 for diabetic retinopathy; E10.2, E11.2, E12.2, E13.2 and E14.2 for diabetic nephropathy; E10.4, E11.4, E12.4, E13.4 and E14.4 for diabetic neuropathy (24, 25). Based on Macular Edema Disease Severity Scale, patients with moderate to severe maculopathy who required laser therapy were also considered as patients with retinopathy (26, 27).

### Statistical analysis

To statistically analyze the recorded data, version 25 of SPSS software was employed. Kolmogorov–Smirnov and Shapiro-Wilk normality tests, P-P plot, and histograms were used to test for the normality of the study population. The tested variables were discovered to be normal and the null hypothesis was rejected. Univariable analysis of potential categorical and continuous risk factor variables was performed using t-test and chi-square test, respectively. Mean ± standard deviation (SD) was used to report continuous, and proportions were used to report values for categorical variables. Logistic regression was conducted to ascertain the effects of NAFLD, liver enzymes and FIB-4 on microvascular complications and APRI on NAFLD. The results were adjusted for age, sex, and duration of diabetes, 2hpp, FBS, Cr, and BMI. Multifocal logistic regression and 4 models group of covariates used for evaluation of relationship between NAFLD and diabetic microvascular complications. A p-value < 0.05 was considered statistically significant.

## Results

### Characteristics of the study population

The level of ALKP, liver enzymes (ALT, AST, GGT) as well as the presence of non-alcoholic fatty liver based on the ultrasound findings were assessed and statistically analyzed as possible predictors of microvascular complications. The baseline characteristics of the study population were summarized in **Table 1**. Overall, the patients with fatty liver tended to be significantly younger compared to those without (Age: 51.34 ± 12.86 vs. 57.54 ± 28.91, p-value<0.001), have lower levels of FBS, SBP, shorter drug duration and HDL, and BMI, DBP, cholesterol (Chl), LDL, Tg, and uric acid (UA) (**Table 1**). Moreover, 17.62% and 8.67% of the NAFLD patients had neuropathy and nephropathy, respectively, which were significantly higher than patients without NAFLD (p-value: 0.015 and p-value: 0.022, respectively). However, the prevalence of retinopathy incidence was not significant between the patients with and without NAFLD.

### Association of gender and baseline characteristics and microvascular complications of diabetes

Considering the importance of the role of gender in the occurrence of non-alcoholic fatty liver, statistical analysis was performed to investigate the role of gender in NAFLD determinants. In the group of fatty liver, females were older significantly (52.02 ± 12.98 vs. 50.56 ± 12.70, p-value=0.005), had lower SBP, DBP, drug duration, FBS, 2hpp, HbA1C, TG, Cr, uric acid, AST, ALT and higher Chol, HDL, LDL, BMI and ALKP, although there were no statistically significant in the prevalence of microvascular complications in fatty liver group based on gender (**Table 1**).

### Association of serum liver enzymes and NAFLD with microvascular complications of diabetes

**Tables 2** summarizes the association between liver enzyme levels and incidence of the three outlined complications. ALKP increased the incidence risk of diabetic neuropathy and nephropathy (Odds ratio (OR):1.002 (95% confidence interval (CI) 1.001-1.003), and 1.002 (1.001-1.004), respectively). Moreover, GGT was a risk factor for the incidence risk of diabetic nephropathy (1.006 (1.002-1.009)). AST and ALT were inversely associated with the risk of diabetic retinopathy (0.989 (0.979-0.998) and 0.990 (0.983-0.996), respectively). Tertiary multivariate adjusted model was used to adjust for duration of diabetes, fasting blood sugar level, sex, age, 2hpp, creatinine, and BMI. As shown in **Table 3**, different parameters were used in 3 models to investigate the relationship between NAFLD and microvascular complications of type 2 diabetes. The results of these models are in general agreement with the baseline model and indicate that the incidence risk of diabetic neuropathy and nephropathy were significantly increased in patients with NAFLD in base line model and even after adjusting for various confounding variables (OR: 1.338 (95% CI: 1.091-1.640) and 1.333 (1.007-1.764), respectively). On the contrary, the incidence of the other complication, retinopathy, was not found to be significantly associated with the presence of NAFLD in patients.

**Table 2:** Association between incidence of diabetic microvascular complications with liver enzymes.

	Retinopathy			Neuropathy			Nephropathy		
	Odds ratio	95% CI	P-value	Odds ratio	95% CI	P-value	Odds ratio	95% CI	P-value
**AST**	0.989	0.979-0.998	0.02	1.000	0.994-1.006	0.993	1.001	0.992-1.011	0.794
**ALT**	0.990	0.983-0.996	0.003	0.995	0.989-1.001	0.079	1.001	0.994-1.009	0.769
**ALK-P**	1.001	1.000-1.003	0.079	1.002	1.001-1.003	0.001	1.002	1.001-1.004	0.008
**GGT**	1.002	0.999-1.005	0.267	1.000	0.997-1.004	0.926	1.006	1.002-1.009	0.001

Data was adjusted for age, sex, duration of diabetes, fasting blood sugar, 2hPP, Cr, and BMI in the tertiary multivariate adjusted model. ALT, alanine aminotransferase; AST, aspartate aminotransferase; ALKP, alkaline phosphatase; GGT, gamma glutamyl transferase.

**Table 3:** Association between NAFLD and diabetes-related microvascular complications among patients with type 2 diabetes [OR 95%CI].

	Model 1	Model 2	Model 3
**Retinopathy**	0.799 (0.624-1.023)	0.848 (0.660-1.089)	0.899 (0.713-1.134)
**Neuropathy**	1.492 (1.199-1.856)	1.540 (1.234-1.923)	1.338 (1.091-1.640)
**Nephropathy**	1.290 (0.963-1.726)	1.253 (0.933-1.684)	1.333 (1.007-1.764)

Model 1: Baseline model.

Model 2: Model adjusted for age and sex.

Model 3: Model adjusted for gender, age, diabetes duration, FBS, 2hPP, Cr, and BMI

NAFLD, non-alcoholic fatty liver disease.

### Association of APRI and NAFLD

**Table 4** summarizes the association between APRI and NAFLD. ARPI_T(1), ARPI_T(2), and ARPI_T(3) values were significantly increased in patients with NAFLD after adjusting for confounding variables (1.440 (1.061-1.954), 1.589 (1.163-2.171), and 2.673 (1.925, 3.710), respectively). Tertiary multivariate adjusted model was used to adjust for duration of diabetes, fasting blood sugar level, sex, age, 2hpp, creatinine, and BMI.

**Table 4:** Association between incidence of NAFLD and APRI.

	NAFLD		
	Odds ratio	95% CI	P-value
**APRI_T(1)**	1.440	1.061-1.954	0.019
**APRI_T(2)**	1.589	1.163-2.171	0.004
**APRI_T(3)**	2.673	1.925-3.710	0.001

Data was adjusted for age, sex, duration of diabetes, fasting blood sugar, and 2hPP in the tertiary multivariate adjusted model. T, tertile. APRI, aspartate aminotransferase to platelet ratio index. NAFLD, non-alcoholic fatty liver disease.

### Association of FIB_4 and microvascular complications

**Table 5** shows the relationship between the severity of liver fibrosis (measured through the FIB-4 index) and the occurrence of microvascular complications of diabetes. In the third tertile of FIB-4 compared to the first one, the risk of complications such as retinopathy and neuropathy is higher (1.116 (0.741-1.681) compared to 1.399 (0.335-5.168) and 1.003 (0.683-1.473) compared to 1.399 (0.371-5.276), respectively; while this trend is reversed in nephropathy (1.087 (0.678-1.744) compared to 0.741 (0.192-2.86). However, the mentioned findings are not statistically significant.

**Table 5:** Association between incidence of microvascular complications and FIB-4.

	Retinopathy			Neuropathy			Nephropathy		
	Odds ratio	95% CI	P-value	Odds ratio	95% CI	P-value	Odds ratio	95% CI	P-value
**FIB-4_T1**	Reference	–	–	Reference	–	–	Reference	–	–
**FIB-4_T2**	1.116	0.741-1.681	0.600	1.003	0.683-1.473	0.988	1.087	0.678-1.744	0.728
**FIB-4_T3**	1.399	0.335-5.168	0.695	1.399	0.371-5.276	0.620	0.741	0.192-2.860	0.741

Data was adjusted for age, sex, duration of diabetes, fasting blood sugar, and 2hPP in the multimodal logistic regression. FIB4, Fibrosis 4 score; T, Tertile.

## Discussion

This prospective cohort study investigated the association of NAFLD and liver enzymes with the incidence of microvascular complications (retinopathy, nephropathy, and neuropathy). After adjustment for confounding factors, NAFLD was a precipitating factor of nephropathy and neuropathy in patients with type 2 diabetes. On the contrary, NAFLD was not a risk factor of retinopathy in patients with type 2 diabetes after adjustment for confounding factors. Although it was found that alkaline-phosphatase increases the incidence risk of diabetic neuropathy and nephropathy and GGT is an increasing risk factor for the incidence risk of diabetic nephropathy. However, other liver enzymes as well as FIB-4 levels were not associated with microvascular complications.

We observed that the incidence risk of diabetic neuropathy is significantly associated with NAFLD. In line with our study, a recent meta-analysis by Greco et al. (28) demonstrated that the prevalence of diabetic neuropathy significantly increased in patients with type 2 diabetes and NAFLD. Moreover, a recent observational study also conformed the association between diabetic neuropathy and NAFLD by measuring NAFLD fibrosis score and FIB-4 (29). Mantovani et al. (30) also suggested that NAFLD exacerbates hepatic and peripheral insulin resistance, presents with a predisposition to atherogenic dyslipidemia, and results in the activation of several pro-fibrogenic mediators, procoagulant, proinflammatory, and pro-oxidant. This can have a crucial role in neuropathy pathology. For instance, several studies established that atherogenic dyslipidemia can directly promote nerve damage via lipotoxicity of free fatty acids and, indirectly, via free fatty acids which can stimulate a systemic inflammatory cytokine cascade and elevate insulin resistance (31, 32). Many other studies also indicated that pro-inflammatory and pro-oxidant mediators have an essential role in neuropathy pathology (32).

Our results showed that the incidence risk of diabetic nephropathy is significantly correlated with NAFLD. In line with our results Casoinic et al. (33) discovered NAFLD to be positively correlated with microalbuminuria, which is a marker of the early stage of nephropathy in patients with type 2 diabetes. A report from India also revealed an increased prevalence of microvascular complications including nephropathy in patients with type 2 diabetes who had fatty liver disease (10). Wen et al. reported that the presence of kidney disease and retinopathy was higher in the “indeterminate risk” and “high risk” groups than in the “low risk” group of NAFLD, after adjusting for the same covariates. They also found that the presence of diabetic kidney disease significantly increased with high NAFLD fibrosis score (34). Another study on Iranian population also reported that NAFLD was not found to increase the risk of diabetic nephropathy (35). Furthermore, Targher et al. (12, 36) reported that NAFLD is independently associated with an increased prevalence of chronic kidney disease in patients with type 2 diabetes. Jia et al. (37) reported a positive association between NAFLD and serum uric acid, tumor necrosis factor-α (TNF-α), insulin resistance index, omentin-1, free fatty acids, homocysteine, and visceral fat area. Any of the above factors combined with NAFLD can elevate nephropathy risk in patients with type 2 diabetes. They established that NAFLD patients showed insulin resistance and elevated visceral fat area, which are the usual components of the metabolic syndrome, a crucial contributor to the progression and development of nephropathy (38, 39). Several studies also indicated a positive correlation between insulin resistance and nephropathy (40, 41).

In contrast to our results, a cross-sectional study of the Korean population reported that the prevalence of nephropathy was lower in patients with type 2 diabetes and NAFLD (11). In addition, Afarideh et al. reported that NAFLD was inversely associated with the prevalence of diabetic nephropathy in the Iranian population (42).

In contrast to our findings Lv et al. reported that NAFLD negatively correlated with the risks of nephropathy, retinopathy and neuropathy (43). Moreover, in contrast to several previously conducted studies (11, 12, 43, 44), our study failed to demonstrate any associations between diabetic retinopathy. However, similar to our results among the Western population and independent of gender, age, ethnicity, serum HDL-C, serum triglycerides, waist circumference, SBP, and A1C, NAFLD was found not to be associated with the presence of retinopathy in the US general population with or without diabetes (45).

These discrepancies in the findings of the studies might be attributed to differences in baseline characteristics of the participants. Furthermore, the ethnic differences for pathophysiological characteristics of patients with type 2 diabetes might also be responsible for the differences between our findings and those of the mentioned studies.

Our result showed ALKP had a significant association with incidence of neuropathy and nephropathy. Also, GGT had a significant association with nephropathy. An inverse association between ALT and AST were also observed with incidence of diabetic retinopathy. Similarly, Afarideh et al. (42), established that ALT had an inverse association with diabetic neuropathy and retinopathy. Similar to our result, a retrospective study reported that elevated ALKP level is associated with nephropathy in patients with type 2 diabetes (46). Circulating ALKP degrades pyrophosphate, which is an endogenous anti-calcification factor in the arterial wall. So, high levels of ALKP can promote arterial calcification and lead to cardiovascular disease (47). Increased arterial stiffness led to elevated systemic blood pressure in the defective glomerular capillaries, with low resistance, and exacerbated intraglomerular hypertension and hyperfiltration, and eventually, nephrosclerosis (46). Therefore, the ALP-diabetic nephropathy association identified in our study may support the role of arterial calcification in the progression of kidney disease (48). Our study failed to demonstrate associations between the level of other liver enzymes and incidence of microvascular complications; However, a recent study by Kim et al. (29) found that the levels of ALT and AST is higher in diabetic neuropathy in patients with NAFLD. In another study, Lin et al. (49) showed that neuropathy is directly associated with GGT in a Chinese population with diabetes.

Despite the existence of conflicting studies on the relationship between microvascular complications of diabetes and NAFLD, several systematic reviews, meta-analyses and umbrella reviews have been conducted to investigate this relationship. It is concluded that NAFLD is a multi-system disease that does not only affect the liver tissue, but it causes many important complications in several organs and increases the mortality of diabetic patients, which is one of the important reasons for the increase in the mortality of coronary artery disease and also diabetic nephropathy (50). Diabetic neuropathy also increases as one of the complications of diabetes following the occurrence of NAFLD in diabetic patients (28), but Dandan Song’s meta-analysis did not report a relationship between diabetic retinopathy and NAFLD (50), and the results of these complications were in line with our study.

In the present study, APRI had significant association with NAFLD. In line with our results, a recent systematic review reported that APRI risk stratify morbidity and mortality in patients with NAFLD (51). Also a recent cross-sectional study in Iran showed that APRI can significantly detect fibrosis in NAFLD (52). Also, a retrospective cohort study in Canada evaluated the prognostic values of non-invasive diagnostic tests such as APRI against liver histology and hepatic venous pressure gradient (HVPG) in NAFLD patients. Their results showed that APRI can predict outcomes of NAFLD patients and it could be used to monitor, risk stratify, and find targeted interventions (53). Furthermore, a prospective study in Brazil demonstrated that is a very accurate in identifying NAFLD (54).

To the best of our knowledge, this is the first prospective cohort study with a population-based sample in Middle East and North Africa (MENA) region to identify the association of NAFLD and liver enzymes with the incidence risk of microvascular complications (retinopathy, neuropathy, and nephropathy) in patients with type 2 diabetes. Another strength of the current study is the sufficient sample size, exact ultrasound grading by single expert operator, as well as the exclusion of other causes of liver disease to assess the presence of NAFLD in patients with type 2 diabetes related microvascular complications.

However, there were some limitations to our study. First, since the present study population was a cohort of patients cared for at a single center, caution should be taken when extrapolating the results to all patients with type 2 diabetes. It should be considered that the majority of the participants were typical patients with type 2 diabetes commonly encountered in outpatient clinics and the present study granted a high degree of consistency regarding, ultrasonographic findings, laboratory data, and the assessment of microvascular diabetic complications. Second, estimated GFR was used rather than a more precise measure of kidney function, e.g., iothalamate clearance. Third, due to the NAFLD definition in the present study based on definite signs of hepatic steatosis (grade 3 hepatic steatosis on abdominal ultrasound), our results may not apply to patients with earlier hepatic steatosis stages on ultrasound or those individuals with sonographically undetectable NAFLD. Fourth, in our database one of the ignored data was consumption of some oral anti-diabetic agents like pioglitazone, so in the next studies, researchers should consider this issue. Moreover, the lack of a liver biopsy which is the gold standard method for diagnosis of NAFLD as well as differentiating it from non-alcoholic stetohepatitis (NASH) (55) can be deemed as the most significant limitation of the present study. Lack of Vibration-controlled Transient Elastography and fibro scan are also other limitations of the current study. However, due to the aggressive nature of liver biopsy, in this study similar to most previous studies, ultrasound was preferred for the diagnosis of NAFLD.

## Conclusion

The present prospective cohort study found that NAFLD, as diagnosed by characteristic sonographic features, was associated with an increased incidence of diabetic nephropathy and neuropathy. Additionally, according to our data, ALKP, GGT, were associated with increased risks of microvascular complication of diabetes, while ALT and AST were shown to be inversely associated with the incidence of diabetic retinopathy. Future studies are required to assess possible mechanisms related to the underlying pathophysiological basis of these associations.

## Data availability statement

The raw data supporting the conclusions of this article will be made available by the authors, without undue reservation.

## Ethics statement

The studies involving human participants were reviewed and approved by Ethics Committee of Tehran University of Medical Sciences. The patients/participants provided their written informed consent to participate in this study.

## Author contributions

ND, FD, and FM performed the data analysis; AE conceived the article; NY, AP, MD, and MP drafted the manuscript; SR and IK provided manuscript revisions; and MN clinically revised manuscript. All authors contributed to the article and approved the submitted version.
